# Purinergic signaling in dengue infection: modulation of P2 receptors as a potential therapeutic strategy

**DOI:** 10.1007/s11302-026-10149-3

**Published:** 2026-04-02

**Authors:** Vitória C. de Melo, Kayla I. de Oliveira, Marina Kipper, Margarete D. Bagatini, Gilnei B. da Silva

**Affiliations:** 1 Postgraduate Program in Biomedical Science, Universidade Federal da Fronteira Sul, Chapecó, SC Brazil; 2 Multicentric Postgraduate Program in Biochemistry and Molecular Biology, State University of Santa Catarina, Lages, Brazil

**Keywords:** Dengue, Purinergic signaling, Immune response, Inflammation

## Abstract

Dengue is a globally widespread arboviral disease characterized by an intense inflammatory response that may progress to severe clinical manifestations, such as dengue shock syndrome and hemorrhagic fever. The lack of effective treatments underscores the urgent need to identify new therapeutic targets. In this context, purinergic signaling—mediated by molecules such as ATP and adenosine—has emerged as a central regulator of immune, inflammatory, and vascular responses during dengue virus (DENV) infection. Activation of purinergic receptors, particularly P2X7, directly influences viral replication, cytokine release, and endothelial barrier integrity, potentially contributing either to viral control or to disease severity when dysregulated. This review synthesizes the main mechanisms by which the purinergic system contributes to dengue pathogenesis, emphasizing both its physiological and pathological roles. The molecular analysis indicates that purinergic signaling plays a pivotal role in both restricting and exacerbating viral infection. Therefore, its pharmacological modulation represents a promising avenue for developing innovative therapeutic strategies, particularly for preventing severe dengue outcomes.

## Introduction

Dengue is an acute, febrile, self-limiting systemic viral infection transmitted by the *Aedes aegypti* and *Aedes albopictus* mosquitoes and is considered a major public health problem in several regions of the world [65]. The etiological agent of dengue is an arbovirus of the genus *Flavivirus* and the family Flaviviridae, classified into four antigenically distinct serotypes (DENV-1, DENV-2, DENV-3, and DENV-4), responsible for infections with different clinical manifestations [44]. Members of this group are enveloped viruses that carry a positive-sense single-stranded RNA genome (Bollati et al., 2010). The dengue virus (DENV) genome is approximately 11 kilobases long and encodes a total of ten proteins, three of which are structural capsid (C), premembrane (prM), and envelope (E), and seven nonstructural (NS1 to NS5) [56].

The biological efficiency of these viral serotypes, combined with the environmental adaptability of their vectors, has facilitated the global expansion of the disease. Between 2000 and 2019, annual symptomatic dengue infections averaged approximately 49 million. However, global estimates have risen due to climate change and urban expansion, leading to wider geographical spread and putting more populations at risk. Consequently, DENV infection estimates for the 2041–2060 period reach 76.9 million, a 56.9% increase over the 2000–2019 period [48].

Clinical manifestations vary broadly, from mild febrile illness to severe and potentially life-threatening conditions such as dengue hemorrhagic fever (DHF) and dengue shock syndrome (DSS), which are associated with increased morbidity and mortality [65]. Although the case fatality rate for dengue fever is generally less than 1%, the infection can progress to severe clinical forms, such as dengue hemorrhagic fever. When diagnosed early and appropriate treatment is instituted, the case fatality rate remains between 2 and 5%. However, in the absence of proper management, this rate can reach up to 20% [51].

Although several studies are exploring pharmacological alternatives, no antiviral drugs are approved for the treatment of dengue; therefore, clinical management focuses on supportive measures and adequate hydration. Symptomatic treatment is used to relieve fever, myalgia, and fatigue (Sinha et al., 2024). Laboratory confirmation can be obtained through various diagnostic approaches, such as viral isolation, antigen detection, PCR, or serological assays (Schaefer et al., 2025). Vaccine development has been an important focus of dengue research. The approval of Dengvaxia in 2015 represented a milestone in prevention, and other candidates, such as Denvax, are currently being tested in clinical trials in Latin America and Asia [54].

The vector competence of *Aedes aegypti* for the Dengue virus (DENV) is modulated by a complex network of cellular signaling that orchestrates the host’s immune responses. Recent literature emphasizes that the mosquito’s innate immunity is primarily coordinated by three conserved signaling pathways: Toll, immunodeficiency (IMD), and JAK/STAT (Janus kinase/signal transducer and activator of transcription) [72].

Inflammation is a key driver of dengue pathogenesis, initiated by the activation of immune cells through Toll-like receptors and subsequent release of proinflammatory cytokines such as TNF-α and IL-6 [34]. Although essential for antiviral defense, excessive cytokine production leads to endothelial dysfunction and increased vascular permeability, contributing to disease severity [4]. In this setting, purinergic signaling plays a modulatory role: Extracellular ATP released from infected cells activates purinergic receptors, particularly P2X7, which amplifies inflammatory responses and vascular damage and also influences viral replication and viral load [19, 20].

The aim of this study was to analyze the pathophysiology of dengue fever, with emphasis on the mechanisms involved in purinergic signaling. We sought to understand the role of this system in regulating the immune response and modulating inflammation and to investigate how activation of purinergic receptors can affect viral replication and vascular complications associated with the infection.

## Dengue: an overview

### Genetic and structural features of dengue virus

The dengue virus is composed of a single-stranded positive-sense RNA genome, surrounded by a lipid bilayer. From this, a polyprotein is generated that is cleaved into ten proteins. Its structure is defined by capsid, premembrane, and envelope proteins, which are critical for assembly and infectivity. Additionally, seven nonstructural proteins are essential for the virus’s vital functions. The nonstructural proteins play roles in viral RNA replication, immune evasion, and control of the host’s cellular response [76].

The dengue virion (DENV) is a spherical particle with an icosahedral structure that can develop into two forms: immature and mature. The virion is coated by a lipid membrane that originates from the host cell’s endoplasmic reticulum. Within this outer layer are the glycoproteins E and M. Internally, the viral RNA genome forms the viral core by binding to the capsid protein. After this, the M and E proteins must undergo structural changes for DENV to become infectious. However, successful viral maturation depends on environmental acidity [84].

The NS1 protein is essential for viral replication and immune evasion. Immune evasion occurs by suppressing the host’s complement system. Consequently, serum NS1 protein levels in the host enable early diagnosis of DENV and serve as a reliable biomarker. Furthermore, the NS2A and NS4B proteins are crucial because they enable the virus to establish itself in the host organism [84].

### Host immune response and immunopathogenesis of dengue

The immune system is essential in the pathogenesis of DENV infection. Therefore, the innate immune response plays a central role in the early stages of the disease. When the adaptive immune response begins, T cells initially provide protection, but they can later evolve into a pathogenic response. Therefore, the initial T helper 1 cell response is essential to combat DENV. However, a later T helper 2 cell response is very dangerous, as it is associated with exaggerated inflammatory reactions. Such an immune imbalance may culminate in a cytokine storm, which amplifies disease severity [58, 99, 105].

Vector-derived factors also influence disease progression. Mosquito saliva modulates host immunity by inhibiting interferon signaling, enhancing viral replication, promoting inflammation, and impairing coagulation, thereby exacerbating disease manifestations [99]. In contrast, asymptomatic individuals often display increased activation of natural killer cells and cytotoxic T lymphocytes, suggesting that effective cytotoxic responses are critical for limiting disease development [31].

Severe dengue manifestations, including hemorrhagic fever, are more frequently associated with secondary infections. This phenomenon is largely attributed to antibody-dependent enhancement, whereby nonneutralizing antibodies facilitate viral entry into host cells, increase viral replication, and amplify inflammatory cytokine release, ultimately leading to vascular leakage [105].

### Cytokines and inflammatory mediators in dengue severity

**Fig. 1:**
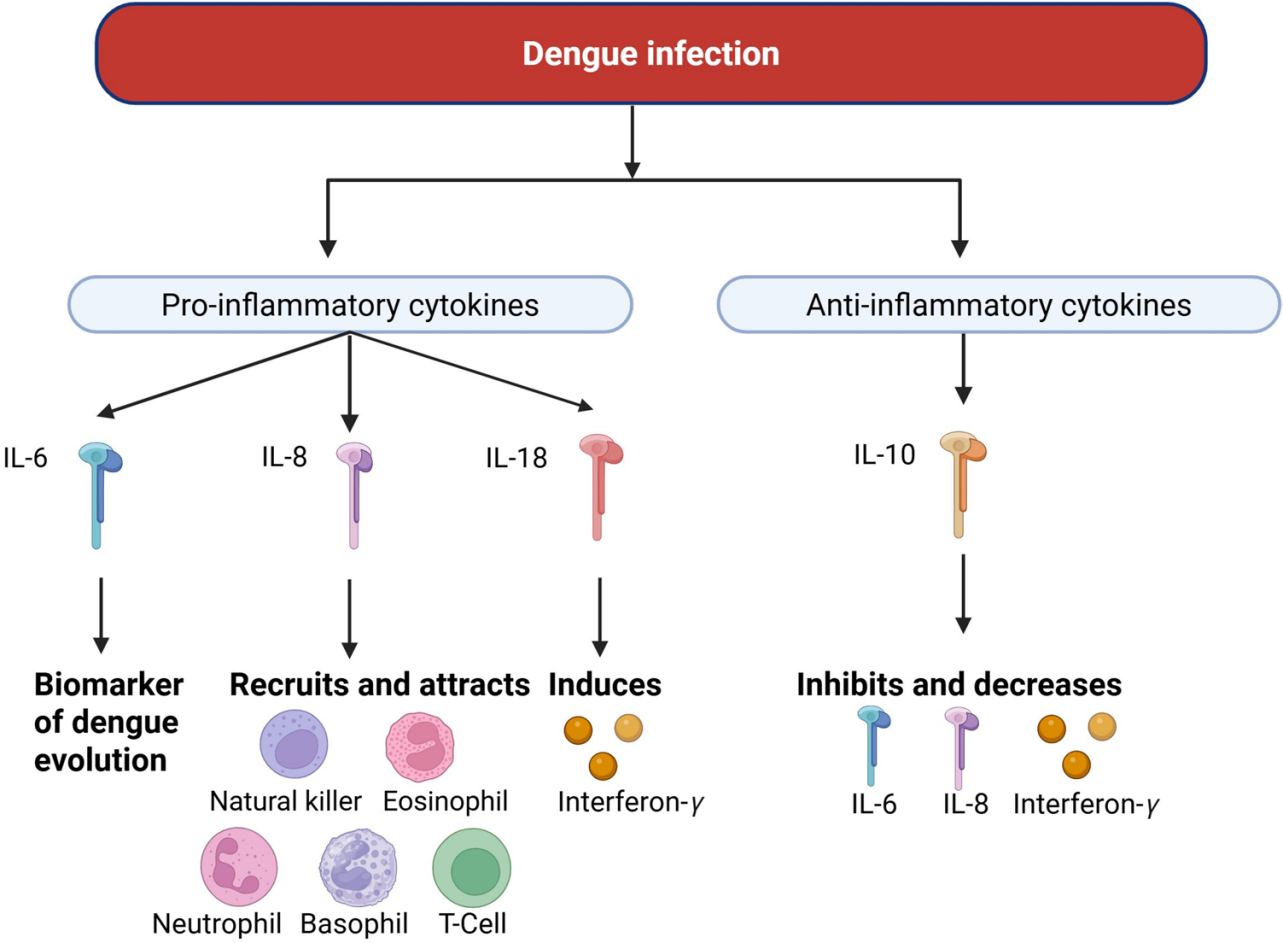
Cytokines found at elevated levels in DENV infection. The figure illustrates the release of key cytokines found at elevated levels during dengue virus (DENV) infection. Among them, IL-6, IL-8, and IL-18 are proinflammatory, while IL-10 plays an anti-inflammatory role. IL-6 is a pivotal inflammatory mediator that may serve as a potential biomarker for monitoring disease progression. IL-8 functions primarily in neutrophil recruitment and also attracts natural killer cells, T lymphocytes, basophils, and eosinophils via chemotactic signaling. Its elevated presence has been associated with pleural effusion. IL-18 promotes the production of interferon-γ, further amplifying the inflammatory response. In contrast, IL-10 counteracts inflammation by suppressing IL-6, IL-8, and interferon-γ production

DENV infection is characterized by elevated levels of multiple cytokines, including IL-6, IL-8, IL-10, and IL-18 (Fig. 1). While these mediators are essential for immune regulation, their overproduction contributes significantly to pathology [96]. Increased concentrations of IL-6, IL-10, and TNF-α have been consistently associated with severe dengue, highlighting the central role of dysregulated inflammation in disease pathogenesis (Solórzano et al., 2021; [99]). Furthermore, variations in IL-6 and IL-8 levels have been linked to distinct clinical presentations [32].

Regarding specific mechanisms, IL-6 is a critical cytokine for maintaining homeostasis through tissue regeneration and hematopoiesis; however, during DENV infection, its overproduction leads to a hyperinflammatory state (Cowell et al., 2023). IL-6 promotes the activation of the coagulation cascade and induces pyrexia, with its concentration being directly proportional to clinical severity. Moreover, the association between elevated IL-6 levels and thrombocytopenia is a key biomarker of severe manifestations [82] (Cowell et al., 2023).

Similarly, IL-8 plays a pivotal role by promoting the recruitment and activation of neutrophils. This cytokine is a major contributor to vascular permeability and angiogenesis, and its elevated levels have been specifically associated with pleural effusion in dengue patients (Iani et al., 2016). In tandem, IL-1, produced mainly by monocytes and macrophages, induces interferon-γ production and amplifies inflammatory signaling. Although its precise role remains under investigation, elevated IL-18 levels are frequently reported in severe cases [74].

In contrast to these proinflammatory mediators, IL-10 acts as a key immunoregulatory cytokine by suppressing the release of inflammatory mediators and modulating antigen-presenting cell function. Nonetheless, imbalances in IL-10 signaling indicate profound immune dysregulation [104] (Iani et al., 2016). Studies by Puc et al. (2021) demonstrate that IL-10 is an effective biomarker for differentiating dengue from other febrile illnesses. Paradoxically, IL-10 may promote viral proliferation by reducing platelet counts and further dysregulating the host response [82].

Consequently, profiling these interleukins is a crucial mechanism for identifying patients at higher risk of progressing to dengue hemorrhagic fever [52].

### Host immune modulation and inflammatory context in dengue infection

Beyond cytokines, chemokines critically shape dengue immunopathogenesis. Among these mediators, the platelet-derived chemokine CXCL4 has been proposed as a biomarker of DENV infection, with significantly higher plasma levels observed in infected individuals than in uninfected controls. Experimental evidence demonstrates that CXCL4 enhances DENV replication in monocytes by engaging the CXCR3 receptor and activating the p38 MAPK signaling pathway, thereby inhibiting STAT-2 and IRF-9 and suppressing IFN-γ production. This mechanism leads to a three to fourfold increase in viral replication, accompanied by reduced levels of proinflammatory cytokines, including TNF-α, IL-1β, and IL-6. Accordingly, pharmacological blockade of the CXCL4–CXCR3 axis using AMG487 has been associated with improved survival in DENV infection [90]. In addition to CXCL4, elevated levels of other chemokines such as CXCL8/IL-8, CCL2/MCP-1, and CXCL10/IP-10 have been reported during DENV infection, reinforcing the role of chemokine-driven immune modulation in disease progression (Solórzano et al., 2021).

The temporal dynamics of the immune response further contribute to dengue pathogenesis. Cytokine profiling studies have identified an early stage of infection characterized by the presence of DENV RNA and/or NS1 antigen, followed by a late stage marked by viremia and associated with anti-DENV IgM production. The distinction between primary and secondary infections is based on antibody profiles: Primary infection occurs in the absence of anti-DENV IgG, whereas secondary infection is defined by the presence of NS1 antigen or viral RNA alongside anti-DENV IgG [32]. These immunological differences are critical determinants of disease severity and inflammatory burden.

Additional factors influencing dengue immunopathogenesis include prior exposure to related flaviviruses and vaccination status. Dengue vaccines such as Dengvaxia exhibit variable efficacy depending on viral serotype, age, and geographic context and are currently recommended only for seropositive individuals in endemic regions (Hou et al., 2022; [102]). Other vaccine candidates, including those developed by the Butantan Institute and Takeda (TAK-003), show differential protection across DENV serotypes and prior exposure status, underscoring the complexity of immune priming in dengue [53] (Tricou et al., 2024). Moreover, co-circulation of DENV and Zika virus (ZIKV), which share high genetic and structural similarity and activate type I interferon responses, has been shown to influence disease outcomes. While certain secondary infection sequences confer cross-protection, ZIKV infection followed by DENV-2 infection has been associated with increased disease severity (Estofolete et al., 2019; [2], Leite-Aguiar et al., 2024).

### Transmission and replication of the dengue virus

Dengue virus (DENV) transmission occurs primarily through the bite of infected female *Aedes aegypti* mosquitoes, although *Aedes albopictus* and other *Aedes* species also contribute to viral spread, particularly in temperate regions. Following ingestion of viremic blood, DENV infects and replicates in the mosquito midgut epithelial cells and then disseminates to secondary tissues such as the hemocoel and salivary glands [95]. Viral replication in salivary gland acinar cells enables the release of mature infectious virions into the saliva, facilitating efficient transmission to the human host during subsequent blood feeding [72, 75].

In humans, DENV is deposited into the skin along with mosquito saliva, which contains immunomodulatory factors that suppress antiviral interferon responses, promote viral replication, and enhance local inflammation. Early after inoculation, DENV infects resident skin immune cells, including Langerhans cells, dermal dendritic cells, macrophages, and keratinocytes. These cells serve as primary targets for viral replication and act as vehicles for viral dissemination to drain lymph nodes and peripheral tissues [16, 99, 105].

At the cellular level, DENV entry is mediated by receptor-dependent endocytosis, predominantly through clathrin-coated pits. Multiple attachment factors and receptors have been implicated, including DC-SIGN, the mannose receptor, TIM and TAM family receptors, and heparan sulfate, reflecting the virus’s broad cellular tropism. Following endosomal acidification, conformational rearrangements in the viral envelope (E) protein trigger membrane fusion, releasing viral genomic RNA into the cytoplasm [73, 84].

Once in the cytoplasm, the positive-sense single-stranded RNA genome is directly translated into a polyprotein, which is co- and post-translationally cleaved by viral and host proteases into structural and nonstructural proteins. Viral RNA replication occurs in association with rearranged endoplasmic reticulum (ER) membranes, forming replication complexes that shield viral RNA from host immune sensing. Newly synthesized genomes are packaged with capsid protein and bud into the ER lumen, acquiring prM and E proteins. Immature virions undergo further maturation in the trans-Golgi network, where acidic pH induces prM cleavage, yielding fully infectious particles that are released via exocytosis [47, 56, 73].

Importantly, DENV exhibits a marked tropism for immune cells. Monocytes, macrophages, and dendritic cells represent the principal targets of productive infection in vivo and play a central role in viral amplification, cytokine production, and systemic dissemination. Infection of these cells profoundly alters innate immune signaling, promoting the release of proinflammatory cytokines, chemokines, and reactive oxygen species, while simultaneously interfering with antiviral interferon pathways [31, 57]. In secondary infections, antibody-dependent enhancement further facilitates viral entry into Fc receptor–bearing immune cells, leading to increased viral replication and exacerbated inflammatory responses. This phenomenon has been extensively documented in dengue virus infection using multiple in vitro and in vivo experimental models, including macrophage-like cell lines and nonhuman primates (Halstead and O’Rourke, 1977 [70, 71, 103],).

Beyond immune cells, endothelial cells can be infected or indirectly activated by viral proteins and inflammatory mediators. DENV exposure induces endothelial activation and dysfunction, resulting in the secretion of cytokines and chemokines, increased vascular permeability, and apoptotic signaling. These alterations are central to the development of plasma leakage and other severe dengue manifestations, including hypovolemic shock [24, 108].

Collectively, DENV transmission and replication are tightly intertwined with host immune activation. Viral replication within immune and endothelial cells establishes a highly inflammatory microenvironment characterized by cytokine and chemokine imbalance, oxidative stress, cellular damage, and endothelial dysfunction. In this context, the release of endogenous danger-associated molecular patterns (DAMPs), particularly extracellular ATP, is a critical consequence of infection-induced cellular stress. Extracellular ATP acts as a potent immunomodulatory signal that shapes antiviral responses, inflammasome activation, and vascular integrity, thereby linking viral replication dynamics to immune-mediated pathology and disease progression [19, 114].

## Purinergic system in the context of dengue virus infection

Purinergic signaling represents a fundamental mechanism of intercellular communication that becomes particularly relevant under conditions of cellular stress, tissue injury, and infection. In the context of dengue virus (DENV) infection, intense immune activation, endothelial perturbation, and cell damage create a microenvironment characterized by the release of endogenous danger-associated molecular patterns (DAMPs), notably extracellular adenosine 5′-triphosphate (ATP). Once released into the extracellular space, ATP and its metabolites function as potent signaling molecules that modulate immune responses, inflammation, and vascular integrity, processes central to dengue immunopathogenesis [19, 20, 85].

The concept of extracellular purines as signaling mediators emerged from early observations of adenosine-induced physiological effects and ATP release following neural stimulation, challenging the traditional view of ATP solely as an intracellular energy molecule [30, 45]. These findings culminated in the formal definition of purinergic signaling as a distinct biological system mediated by extracellular nucleotides and nucleosides [7]. Subsequent studies established ATP and adenosine as dual-function molecules capable of regulating cellular responses across multiple physiological and pathological contexts, including immune and vascular systems [11, 92].

In addition to ATP, its extracellular hydrolysis products—adenosine diphosphate (ADP), adenosine monophosphate (AMP), and adenosine—also act as biologically active mediators, collectively shaping the magnitude and duration of purinergic signaling responses [12]. The balance between ATP release and its enzymatic degradation is therefore a critical determinant of inflammatory outcomes during viral infections.

An overview of the main purinergic components, target cell types, experimental models, and immunological outcomes described in dengue virus infection is summarized in Table 1.

**Table 1:** Purinergic signaling pathways involved in dengue virus infection. Summary of purinergic components, target cell types, experimental models, and main findings related to immune regulation, antiviral responses, inflammasome activation, and endothelial dysfunction during dengue virus (DENV) infection

Purinergic component	Target cell type(s)	Model	Findings	References
Extracellular ATP (eATP)	Human monocytes	In vitro (DENV-infected monocytes)	eATP stimulation reduces intracellular DENV antigen and NS1 levels, restricting viral replication and modulating proinflammatory cytokine production	[19]
P2X7 receptor (P2X7R)	Monocytes	In vitro	Activation of P2X7R induces ROS and NO production, restricting DENV replication in human monocytes; mechanistic support for P2X7R-mediated antiviral responses is provided by nondengue models	[19, 21]
P2X7 receptor (P2X7R)	Dendritic cells and γδ T cells	In vitro (DC–PBMC co-culture)	Pharmacological inhibition of P2X7R reduces IFN-γ production by γδ T cells, indicating a role in antiviral immune regulation rather than direct control of viral replication	Tsai et al., 2015
Extracellular ATP/P2X7R	Immune cells	In vitro (nondengue infectious models)	P2X7R activation triggers potassium efflux and ROS generation, key signals for NLRP3 inflammasome activation, as demonstrated in nondengue infectious models	[37], Cruz et al., 2007; [17]
NLRP3 inflammasome	Monocytes and macrophages	In vitro and clinical studies	Sustained NLRP3 inflammasome activation promotes IL-1β and IL-18 maturation, contributing to excessive cytokine release and disease severity in dengue	[93], Patro et al., 2019; [110]
Extracellular ATP (eATP)	Endothelial and immune cells	In vitro and translational studies	eATP released during infection acts as a danger-associated molecular pattern (DAMP), activating purinergic receptors and modulating inflammatory and endothelial responses	[27]
Purinergic P2 receptors	Endothelial cells	In vitro (DENV-infected endothelial cells)	Dengue-associated inflammatory mediators disrupt endothelial junction integrity and increase vascular permeability; purinergic signaling is discussed as a potential modulatory framework for these inflammation-driven endothelial responses	Dalrymple et al., 2012; [55]
CD73 (ecto-5′-nucleotidase)	Endothelial cells (HUVECs)	In vitro (DENV-2 infection)	Dengue virus type 2 modulates endothelial barrier function through CD73 activity, implicating the CD73/adenosine pathway in dengue-associated vascular permeability	[27, 115], Patkar et al., 2023
eATP/P2X7R/NLRP3 axis	Immune and endothelial cells	Clinical observations	Patients with severe dengue exhibit elevated extracellular ATP levels and enhanced activation of the P2X7R/NLRP3 axis, associated with cytokine storm and severe clinical outcomes	[57, 110]
Adenosine pathway	Endothelial and immune cells	Conceptual and experimental studies	Adenosine signaling modulates inflammatory responses and vascular function, potentially counterbalancing excessive ATP-mediated inflammation during dengue	[27, 115]
Matrix metalloproteinases (MMP-2, MMP-9)	Endothelial cells	In vitro (DC–endothelial interaction)	DENV-infected dendritic cells release MMPs that downregulate VE-cadherin expression, increasing endothelial monolayer permeability	[66]

### Purinergic receptors and extracellular nucleotide metabolism

The biological effects of extracellular purines are mediated through membrane-bound purinergic receptors, which are widely expressed across immune, endothelial, and stromal cells. These receptors are classified into two main families: the P1 group, activated by the nucleoside adenosine, and the P2 group, which interacts with extracellular nucleotides including purines such as ATP/ADP and pyrimidines like UTP/UDP [39, 112].

P1 receptors include four G protein-coupled receptor (GPCR) subtypes: A1, A2A, A2B, and A3. These seven-transmembrane domain proteins exhibit diverse tissue distributions and distinct functional profiles [38]. A1 and A3 receptors couple to Gi/o proteins, thereby inhibiting adenylate cyclase and reducing intracellular cyclic AMP (cAMP) levels. Conversely, A2A and A2B receptors couple to Gs proteins, leading to upregulation of cAMP production [26, 98].

P2 receptors are subdivided into ionotropic P2X and metabotropic P2Y subfamilies. P2X receptors (P2X1–P2X7) function as ATP-gated ion channels that mediate rapid ionic fluxes, particularly of calcium, sodium, and potassium, thereby initiating downstream inflammatory signaling cascades [116]. In contrast, P2Y receptors are G protein-coupled receptors that activate intracellular signaling pathways via Gq or Gi proteins, depending on the subtype. Notably, P2Y11 uniquely couples to both Gq and Gs proteins, enabling simultaneous modulation of calcium and cAMP signaling [9, 14].

Extracellular nucleotide concentrations are tightly regulated by ectonucleotidases expressed on the cell surface. CD39 catalyzes the sequential hydrolysis of ATP and ADP to AMP, while CD73 converts AMP into adenosine, thereby shifting signaling from proinflammatory ATP-mediated responses toward generally immunoregulatory adenosine signaling [22, 115]. Dysregulation of this enzymatic axis has significant implications for inflammatory diseases, including viral infections.

### Immunological and vascular functions of purinergic signaling

Purinergic signaling plays a central role in immune regulation by controlling leukocyte activation, cytokine release, cell migration, and inflammasome activation. ATP released from stressed or damaged cells acts as a potent immunostimulatory signal, amplifying innate immune responses by engaging P2 receptors on monocytes, macrophages, dendritic cells, and lymphocytes [8, 25].

Purinergic signaling is a critical determinant of endothelial function and permeability within the vasculature. ATP exerts a dual effect on vascular tone: It triggers vasoconstriction via P2X receptor activation on smooth muscle cells, whereas endothelial-derived ATP stimulates P2Y receptors to facilitate nitric oxide–mediated vasodilation [3, 8]. Importantly, the P2X7 receptor is expressed within the endothelium and plays a pivotal role in driving oxidative stress, compromising barrier integrity, and triggering apoptosis. These mechanisms are central to the development of vascular leakage syndromes [89]. These deleterious effects are attributed to P2X7-mediated pore formation and NADPH oxidase-driven ROS production (Shibata et al., 2018).

Platelets represent another key interface between purinergic signaling, inflammation, and vascular pathology [34]. ADP-mediated activation of P2Y12 receptors drives platelet aggregation and hemostatic responses, while platelet-derived mediators also modulate innate and adaptive immune functions. These mechanisms are particularly relevant in dengue, where immune activation and hemostatic imbalance coexist [20].

### Purinergic modulation of immune responses during dengue virus infection

The purinergic system plays an important role in modulating immune responses during dengue virus (DENV) infection by regulating antiviral and inflammatory signaling. Although DENV can infect multiple cell types in vitro, immune cells, particularly dendritic cells, monocytes, and macrophages, are the primary targets of viral replication in vivo, where they contribute to viral dissemination, inflammation, and disease pathogenesis. Purinergic signaling in these cells influences cytokine release and immune activation during dengue infection [57, 87].

The recognition of DENV-derived pathogen-associated molecular patterns (PAMPs) by pattern recognition receptors, such as Toll-like receptors (TLRs), triggers inflammatory signaling pathways. This process promotes robust cytokine production and cellular stress responses, which are further amplified by the infection-induced release of extracellular ATP and subsequent inflammasome activation (Lee et al., ). As a consequence of infection-induced stress or cell damage, extracellular ATP (eATP) is released into the tissue microenvironment, where it acts as a damage-associated molecular pattern (DAMP) capable of recruiting immune cells and activating purinergic P2 receptors expressed on these cells [13, 27].

Among purinergic receptors, P2X7 has been extensively studied in the context of DENV infection. This receptor is highly expressed on immune cells and becomes activated under inflammatory conditions characterized by elevated extracellular ATP concentrations (Lister et al., 2007). Upon activation, the P2X7 receptor triggers calcium influx, potassium efflux, and the production of reactive oxygen species (ROS), processes that orchestrate NLRP3 inflammasome activation and regulate inflammatory and antiviral responses [80, 113].

Experimental studies using DENV-2–infected human monocytes have demonstrated that extracellular ATP activates P2X7, reducing viral replication and decreasing levels of the viral NS1 protein, which is essential for DENV replication [19, 21]. Pharmacological stimulation of monocytes with ATP prior to infection inhibits DENV-2 replication, whereas selective inhibition of P2X7 abolishes this antiviral effect, indicating a direct involvement of this receptor in controlling viral infection [19].

In addition to its antiviral effects, P2X7 activation modulates cytokine and chemokine production during DENV infection. ATP-stimulated monocytes exhibit reduced secretion of inflammatory mediators, including CCL2, CXCL10, IL-8, and TNF-α, alongside increased production of NO and ROS [19]. These findings suggest that P2X7 activation supports mechanisms that contribute to both viral control and regulation of the inflammatory response, although the precise mechanisms remain incompletely understood.

Purinergic signaling also influences adaptive immune responses during dengue infection. In dendritic cells, inhibition of P2X7 has been shown to markedly reduce interferon-γ (IFN-γ) production by γδ T cells, indicating that P2X7-mediated signaling in antigen-presenting cells contributes to antiviral T cell responses (Tsai et al., 2015). Together, these data support a role for P2X7 as a modulator of both innate and adaptive immunity during DENV infection.

Collectively, these findings indicate that P2X7 activation contributes to viral clearance during DENV infection through converging mechanisms. By inducing ROS and nitric oxide production, P2X7 creates an intracellular environment that is unfavorable for viral replication and compromises the stability and function of viral proteins such as NS1, thereby disrupting the formation of replication complexes [19]. In parallel, P2X7 signaling may limit the pool of infected cells through regulated cell death pathways and enhance antiviral adaptive immunity by supporting IFN-γ production in γδ T cells (Tsai et al., 2015). Together, these effects position P2X7 as a central immunological hub linking innate effector responses, adaptive immunity, and direct interference with the DENV life cycle, ultimately contributing to effective control of viral infection.

### Exacerbated purinergic activation and its relationship with dengue severity

**Fig. 2:**
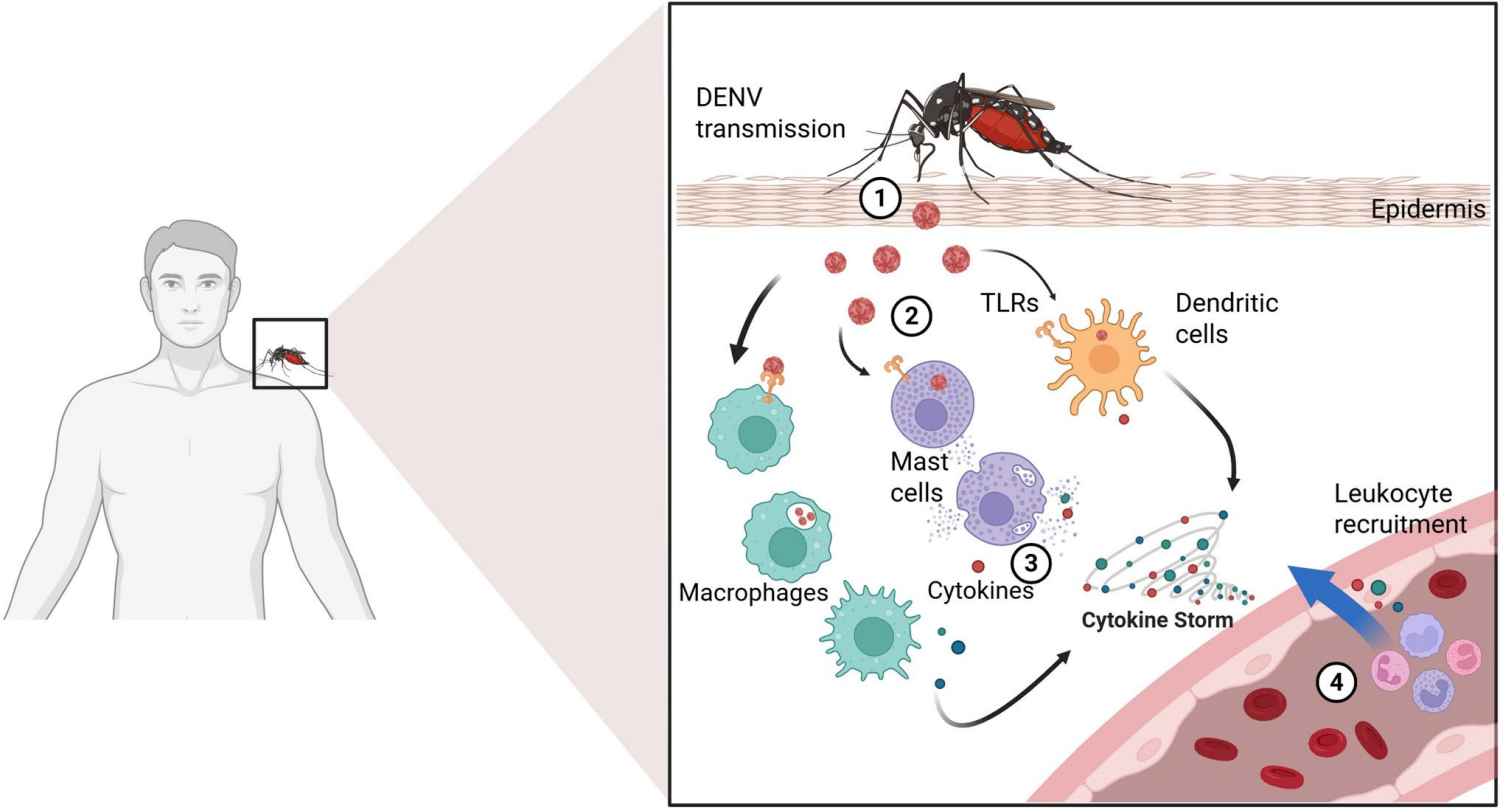
Innate immune response in DENV infection. (1) The *Aedes aegypti* mosquito transmits dengue virus (DENV) during the bite. (2) DENV infects monocytes, macrophages, and dendritic cells, which express Toll-like receptors (TLRs), initiating the innate immune response. (3) Inflammasome activation and extracellular ATP release lead to purinergic signaling activation, amplifying the production of inflammatory cytokines and triggering a cytokine storm. (4) Released cytokines promote the recruitment of additional leukocytes to the infection site, intensifying the inflammatory response and potentially contributing to endothelial damage and increased vascular permeability

As discussed above, purinergic signaling constitutes an essential component of host defense during dengue virus (DENV) infection. Nevertheless, sustained and dysregulated activation of this system shifts its role from protective to pathogenic, contributing to excessive inflammation, endothelial dysfunction, and increased vascular permeability (Fig. 2), hallmarks of severe dengue manifestations, including dengue hemorrhagic fever and dengue shock syndrome (Guzmán and Harris, 2015).

In this context, excessive activation of the P2X7 receptor functions as a critical driver of NLRP3 inflammasome dysregulation. Although inflammasome activation is required for effective innate immune responses, its sustained activation during dengue infection amplifies inflammatory signaling beyond physiological control. Overactivation of the NLRP3 inflammasome leads to uncontrolled release of IL-1β and IL-18, triggering an inflammatory feedback loop that amplifies the production of proinflammatory cytokines such as tumor necrosis factor-alpha (TNF-α), IL-6, soluble TNF receptors 1 and 2 (sTNFR1, sTNFR2), interferon-gamma (IFN-γ), and chemokines like CXCL8, CXCL9, CXCL10, CXCL11, and CCL5, alongside the anti-inflammatory cytokine interleukin-10 (IL-10), fostering a deleterious hyperinflammatory environment [93] (Patro et al., 2019) [77, 110].

Moreover, persistent NLRP3 inflammasome activation can induce inflammatory cell death via pyroptosis, exacerbating tissue injury and releasing additional DAMPs that perpetuate purinergic system activation and systemic inflammation [93] (Patro et al., 2019). Clinical studies have demonstrated that patients with severe dengue present elevated extracellular ATP levels and exacerbated activation of the P2X7R/NLRP3 axis, indicating a central role for this pathway in amplifying the inflammatory response and driving disease progression toward severe forms [57, 110].

**Fig. 3:**
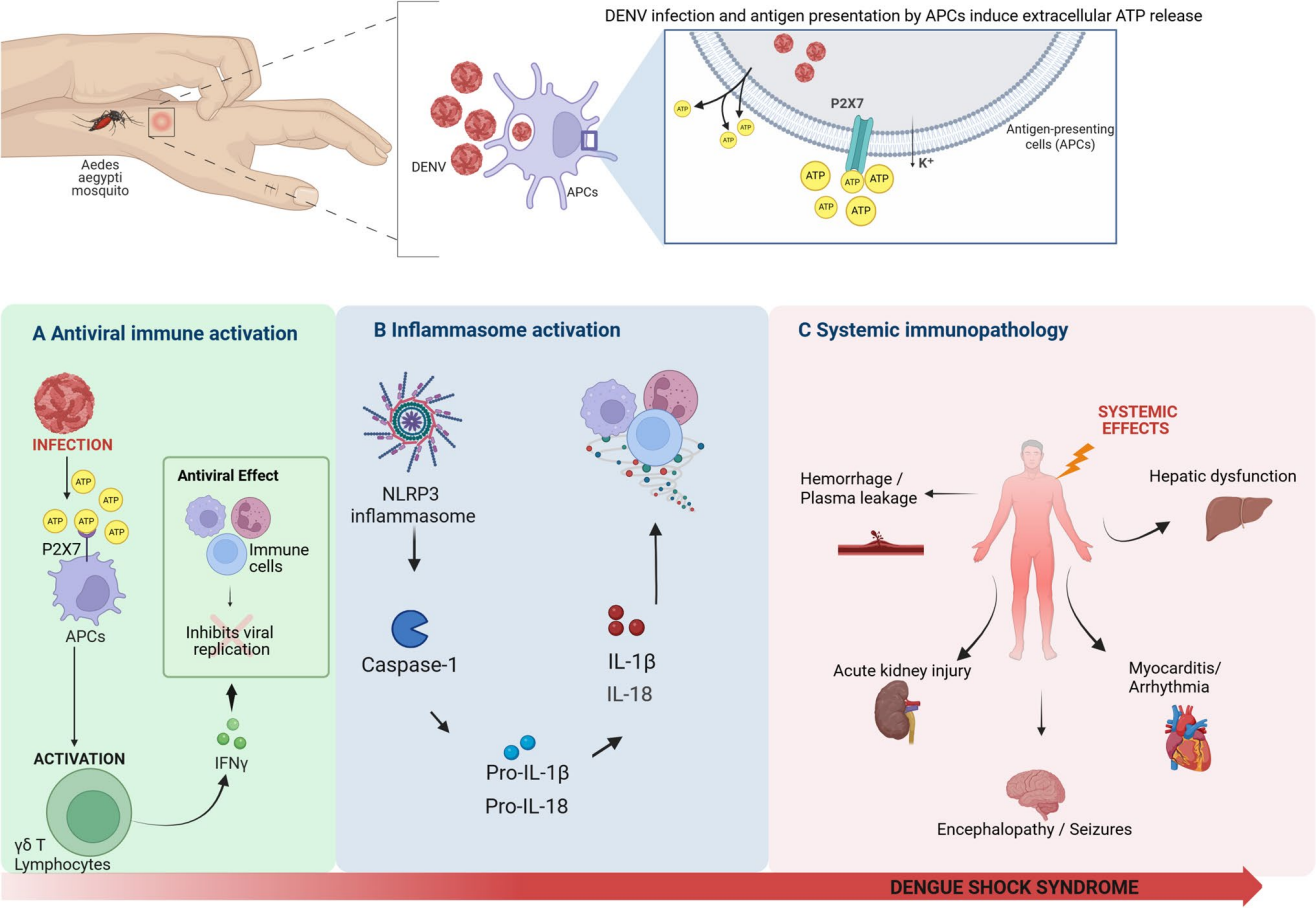
Integrated schematic representation of the immunological mechanisms involved in dengue virus (DENV) infection and disease severity. Following DENV infection and antigen presentation, APCs undergo cellular stress and activation, leading to the release of ATP into the extracellular milieu. Extracellular ATP acts as a danger-associated molecular pattern (DAMP) and binds to the purinergic P2X7 receptor expressed on APCs, triggering downstream immune responses. **A** Antiviral immune activation: Activation of P2X7 on APCs promotes their maturation and enhances the activation of γδ T lymphocytes, resulting in increased production of interferon-gamma (IFN-γ). IFN-γ contributes to antiviral immunity by inhibiting viral replication and amplifying immune effector mechanisms. **B** Inflammasome activation: Sustained stimulation of the P2X7 receptor induces potassium (K⁺) efflux, which promotes the assembly of the NLRP3 inflammasome and subsequent activation of caspase-1. Caspase-1 mediates the processing of pro–IL-1β and pro–IL-18 into their mature, bioactive forms, IL-1β and IL-18, thereby intensifying the proinflammatory response. **C** Systemic immunopathology: While initially protective, excessive or prolonged activation of P2X7-driven inflammatory pathways may become pathogenic, contributing to systemic inflammation characterized by increased vascular permeability, plasma leakage, hemorrhage, and multiorgan dysfunction. Severe clinical manifestations of dengue include hepatic dysfunction, acute kidney injury, myocarditis and/or cardiac arrhythmias, and central nervous system involvement such as encephalopathy and seizures. Collectively, these immune-mediated events contribute to the development of dengue shock syndrome and highlight the ATP–P2X7–inflammasome axis as a potential target for immunomodulatory interventions

This dysregulated increase in inflammatory cytokines, commonly known as a cytokine storm, not only amplifies the innate immune response but is also directly associated with severe clinical complications of dengue, such as plasma leakage, shock, and multiple organ failure (Fig. 3). IL-1β, by upregulating endothelial adhesion molecules, promotes leukocyte recruitment but, at excessive levels, can exacerbate endothelial dysfunction and vascular permeability, leading to edema and hemoconcentration, characteristic features of severe dengue [93, 110].

Collectively, these observations underscore the need for a tightly regulated purinergic response to preserve antiviral immunity while preventing excessive inflammatory amplification that drives severe dengue outcomes.

#### Endothelial dysfunction and increased vascular permeability

Severe dengue is associated with a transient increase in vascular permeability due to endothelial dysfunction during the critical phase of the disease [108]. Vascular leakage typically becomes clinically evident 3–6 days after disease onset, known as the critical phase [67].

The endothelium forms the primary barrier of the circulatory system; thus, dysfunction of endothelial cells during acute illness can affect vascular permeability and cause plasma leakage. In dengue, several factors may contribute to this condition, including endothelial cell apoptosis, the overexpression of chemokines, and monocyte chemoattractant protein-1 (MCP-1) produced by monocytes [62]. In parallel, infection-associated cellular stress and tissue damage lead to the release of extracellular ATP, which acts as a danger-associated molecular pattern (DAMP) capable of activating purinergic receptors expressed on endothelial and immune cells, thereby modulating endothelial barrier integrity [27].

In vitro studies of DENV infection using various cell types have elucidated mechanisms of endothelial dysfunction. DENV-infected endothelial cells secrete IL-6, CXCL10, CXCL11, and RANTES (regulated on activation, normal T cell expressed and secreted) (Dalrymple et al., 2012). These mediators can increase permeability and possess chemoattractant properties, which may contribute to inflammation and plasma leakage in vivo [55]. Dendritic cells infected with DENV produce matrix metalloproteinases (MMP-2 and MMP-9), which increase endothelial monolayer permeability by downregulating VE-cadherin expression [66]. Importantly, experimental and translational studies in vascular biology have demonstrated that extracellular ATP signaling through P2 purinergic receptors can amplify inflammatory responses and modulate endothelial junctional stability, indirectly enhancing cytokine- and MMP-mediated disruption of endothelial barriers under inflammatory conditions [83, 107].

During the acute infection phase, patients exhibit elevated plasma levels of TNF, interferon-gamma (IFN-γ), and macrophage migration inhibitory factor (MIF). Additionally, there is an increase in interferon gamma-induced protein 10 (CXCL10/IP-10) [35]. These inflammatory mediators can further sensitize endothelial cells to purinergic signaling, particularly under conditions of sustained extracellular ATP accumulation [27].

Throughout DENV infection, most immune cells—including monocytes, macrophages, NK cells, invariant natural killer T (iNKT) cells, and DENV-specific CD4 and CD8 T cells—contribute to the increase in TNF-α, which promotes vascular permeability and inflammation [67]. Furthermore, increased interleukin 1 (IL-1) levels contribute to enhanced capillary permeability, and interleukin 6 (IL-6) levels rise in cases of dengue hemorrhagic fever and shock [19]. In vitro studies show that exposure of endothelial cells to TNF-α increases endothelial barrier permeability and induces mitochondria-dependent apoptotic cell death, leading to endothelial dysfunction characterized by oxidative stress and reduced cell viability (Díaz Sanchez et al., 2023).

In this inflammatory environment, activation of the P2X7 receptor by extracellular ATP on immune and endothelial cells may potentiate TNF-α–driven signaling cascades, thereby further compromising endothelial barrier function [19, 107]. Beyond its effects on vascular permeability, TNF-α is a well-established mediator linking inflammation and coagulation in systemic inflammatory states, where it promotes a procoagulant endothelial phenotype by inducing tissue factor expression and suppressing anticoagulant pathways [106]. Although direct evidence for this mechanism in dengue remains limited, elevated TNF-α levels have consistently been reported in patients with severe dengue compared to those with milder disease, reinforcing its association with disease progression [68, 109].

In addition to inflammatory cytokines, purinergic metabolism plays a critical role in regulating endothelial barrier homeostasis. Modulation of CD73 expression, an ecto-5′-nucleotidase, has been shown to influence endothelial permeability during DENV infection. Experimental studies in human umbilical vein endothelial cells (HUVECs) infected with DENV-2 demonstrate an initial increase in CD73 expression mediated by type I interferon, followed by a subsequent reduction that correlates with loss of endothelial barrier integrity. CD73 hydrolyzes AMP to generate adenosine, which acts via P1 purinergic receptors to preserve endothelial junctional stability. Consequently, reduced CD73 expression and diminished adenosine availability contribute to sustained endothelial activation, hypersensitivity, and progression toward dengue hemorrhagic fever [78].

Clinically, increased vascular permeability leads to plasma leakage, fluid accumulation in the pleural and peritoneal cavities, decreased blood pressure and pulse pressure, and subsequent organ hypoperfusion [61, 108]. Postcapillary venules represent the primary sites of increased permeability during dengue-associated vascular leakage [94]. In patients who develop plasma leakage during the critical phase, microvascular circulation abnormalities have been documented, leading to impaired blood flow and tissue perfusion [108].

## Therapeutic implications and future perspectives

The purinergic system, with its complexity and central role in various physiological and pathological processes, remains a promising field of investigation, particularly in the context of infectious diseases such as dengue. A deeper understanding of purinergic pathways and their interactions has been opening new perspectives for innovative and more effective treatments, as well as for modulating the immune response during viral infection.

Recent studies have focused on pharmacological modulation of P2X and P2Y receptors, with particular emphasis on the P2X7 receptor due to its central role in inflammasome activation, cytokine release, and cell death. P2X7 is a distinctive member of the P2X receptor family, predominantly expressed by hematopoietic cells, especially monocytes, and activated only at high extracellular ATP concentrations. This activation threshold enables P2X7 to function as a prototypical danger sensor, linking tissue damage and extracellular ATP accumulation to inflammatory signaling. While transient P2X7 activation may contribute to host defense and immune cell survival, sustained or excessive stimulation promotes apoptosis and pyroptosis, underscoring its dual, context-dependent role in inflammation [11, 111] (Fig. 3).

Given these characteristics, P2X7 has attracted considerable interest as a therapeutic target. Multiple classes of antagonists have been developed, including divalent cations, small-molecule P2 receptor antagonists, organic cations, natural product derivatives, monoclonal antibodies, and synthetic compounds [50]. In the context of viral infections, including dengue, inhibition of P2X7 signaling has been shown to attenuate the release of proinflammatory cytokines, suggesting a potential strategy to mitigate excessive inflammation associated with severe disease manifestations such as dengue hemorrhagic fever and shock [19].

Beyond its inflammatory effects, purinergic signaling has been implicated in regulating dengue viral replication. The viral nonstructural protein 1 (NS1), widely used as a diagnostic biomarker, circulates in the bloodstream from the early stages of infection and acts as a cofactor for efficient viral replication [18, 114]. Experimental evidence indicates that extracellular ATP can reduce NS1 levels in DENV-infected cells, supporting the hypothesis that ATP released from damaged cells or platelets may contribute to the elimination of infected monocytes. In line with this, reduced intracellular DENV antigen levels observed in P2X7-expressing monocytes further confirm that ATP signaling through P2X7 constitutes an important negative regulatory pathway for viral persistence [19].

Collectively, in vitro models of DENV infection in human monocytes demonstrate that extracellular ATP-mediated activation of P2X7 leads to decreased NS1 production, reduced viral load, and attenuation of proinflammatory cytokine and chemokine release [1, 19]. These findings suggest that purinergic signaling not only influences viral replication but also shapes the inflammatory microenvironment associated with disease severity.

Beyond monocytes, ATP-mediated signaling affects multiple inflammatory mediators implicated in dengue pathogenesis. ATP and the P2X7 agonist BzATP have been shown to modulate IL-8 and IL-6 production in DENV infection models. Endothelial cells infected with DENV release IL-8, activate the complement system, and may undergo apoptosis, contributing to endothelial dysfunction and increased vascular permeability (Bosch et al., 2002; [69]). Consistently, elevated circulating IL-6 levels are strongly associated with severe dengue manifestations, including dengue hemorrhagic fever and dengue shock syndrome [32]. Together, these observations highlight the capacity of purinergic signaling to modulate a network of inflammatory mediators that act synergistically to drive disease severity.

TNF-α represents another key mediator regulated by P2X7 activation during dengue infection. While TNF-α plays an essential role in antiviral immune signaling, its excessive production contributes to cytokine storm and tissue damage, including neuropathological complications observed in severe dengue. DENV-induced TNF-α release has been shown to be modulated by pretreatment with ATP and BzATP, reinforcing the role of P2X7 in shaping inflammatory outcomes (Donnelly-Roberts et al., 2009; [19]). Furthermore, blockade of P2X7 signaling in DENV-infected dendritic cells reduces IFN-γ–mediated antiviral responses by γδ T cells, underscoring the complex and context-dependent effects of P2X7 modulation on host immunity (Tsai et al., 2015).

Despite strong experimental support for a role of P2X7 signaling in dengue virus infection, it is important to emphasize that no clinical trials to date have specifically evaluated P2X7-targeted therapies in dengue patients. Current evidence largely derives from in vitro studies using human monocytes, dendritic cells, and endothelial cells, as well as from translational insights from other inflammatory and infectious disease models [19] (Carvalho-Barbosa et al., 2023) [97]. Importantly, the dual nature of P2X7 signaling, protective under controlled activation yet deleterious when sustained, represents a major challenge for clinical translation.

Nevertheless, P2X7 has been extensively investigated as a therapeutic target in nondengue clinical settings, including autoimmune diseases, chronic inflammatory disorders, neuroinflammation, and cancer. Several small-molecule P2X7 antagonists, including AZD9056 and CE-224535, have advanced to phase I and II clinical trials, demonstrating acceptable safety profiles and efficacy in modulating excessive inflammatory responses [41] (Grassi et al., ) [97]. These findings are particularly relevant given the central role of cytokine storm and endothelial dysfunction in severe dengue.

From a future clinical perspective, P2X7-targeted interventions in dengue are best envisioned as adjunctive immunomodulatory strategies rather than direct antiviral therapies. Such approaches may be especially valuable during the critical phase of infection, when excessive inflammasome activation, IL-1β release, TNF-α production, and vascular leakage drive disease progression. Careful consideration of treatment timing and dosing will be essential to preserve beneficial antiviral immune responses while preventing pathological inflammation.

Finally, patient stratification based on biomarkers such as extracellular ATP levels, circulating cytokine signatures, or P2X7 expression on immune cells may help identify individuals most likely to benefit from purinergic modulation. Together, these considerations underscore the need for robust in vivo validation and well-designed clinical trials to determine whether targeting the P2X7 axis can be safely and effectively translated into immunoregulatory therapies for dengue infection.

## Concluding remarks

Dengue virus infection represents a significant global public health challenge, especially due to the lack of effective antiviral treatments and the risk of progression to severe disease forms. In this context, purinergic signaling emerges as an important modulator of immune and inflammatory responses, directly influencing viral replication, immune pathway activation, and endothelial barrier integrity.

The P2X7 receptor, in particular, has stood out as a key mediator of proinflammatory responses and viral load control. Although its activation contributes to antiviral defense through the induction of reactive oxygen species, nitric oxide, and cytokines such as IFN-γ, its overactivation can trigger a cytokine storm and compromise vascular homeostasis, favoring the development of complications such as dengue hemorrhagic fever and dengue shock syndrome [17, 37].

Therefore, understanding the mechanisms that regulate purinergic signaling during DENV infection is essential for identifying promising therapeutic targets. Pharmacological modulation of receptors such as P2X7 with specific antagonists may be an effective strategy to mitigate the deleterious effects of exacerbated inflammation without compromising the immune response necessary for viral clearance.

It is thus concluded that the purinergic system not only plays a significant role in the pathophysiology of dengue but also represents an emerging therapeutic pathway, warranting further research to develop safe, targeted interventions for disease control and its severe manifestations.

## Data Availability

The datasets generated during and/or analyzed during the current study are not publicly available but are available from the corresponding author upon reasonable request.
